# Dental Puffing

**Published:** 1859-09

**Authors:** A. Berry


### DENTAL PUFFING.

BY A. BERRY, D. D. S.

THE advertisements of dentists afford a pretty good criterion, in many instances, by which to form an opinion as to their moral and professional standing.

The announcement of a dentist offering his services to the public is sufficient, without the addition that he has supplied himself with the best instruments, availed himself of all the latest improvements, that he can pull and plug teeth the best in the world, and insert artificial teeth, from one to a full set, so as to answer all the purposes of mastication and defy detection. I saw a physician's card in the papers a few years ago, in which he asserted, among other wonderful things, that he could cure fevers,—quite as appropriate as statements like the above by dentists.

A dentist, commencing to practice in the capital of an Eastern State about a year ago, attempted to astonish the natives by an advertisement in the papers, from which I make a few extracts.

"DENTISTRY.

"Dr. ———, Dentist, would respectfully inform the citizens of ——— and vicinity, that having just concluded a term of three years study and practice with Dr. —— of ——, he has fitted elegant and commodious rooms in ———, where, supplied with every modern appliance or invention, with instruments of the latest and most approved patterns, enabling him to warrant all his operations, and guarantee entire satisfaction.

"Dr. —— having at considerable expense purchased the right to use the instruments manufactured and patented by Dr. Branch, of Illinois, for the local application of anæsthesia, is now prepared to extract teeth entirely free from all pain as well as injury.

"In the insertion of artificial teeth we make use of the atmospheric pressure principle, thereby entirely doing away with the old and awkward method of spiral springs.

"Our facilities for the successful prosecution of our business are second to none in the New England States, and in point of skill, success in practice, and satisfaction received, we acknowledge no superior—surrender to no man,"

Meeting with a lady last summer who was sustaining injury from clasps carelessly applied to her teeth, I said, "You had better see Dr. ——," (the preceptor of the young dentist from whose card the above extracts are made,) to which she replied, "I have not a very good opinion of Dr. ——, he comes out every few months with such a puffing circular!" People generally have learned to suspect that dentists who advertise in the style of patent medicine dealers are no better than they should be.

We expect quacks and venders of all sorts of humbugs to publish puffs and certificates *ad nauseam*, but no dentist with proper respect for his profession will resort to such low artifices; and no one who does so deserves any respect or courtesy from his professional brethren.

